# Tumour-infiltrating lymphocytes differ across MammaPrint® classifications in breast cancer

**DOI:** 10.1530/ERC-26-0116

**Published:** 2026-09-15

**Authors:** Carmen Ariño-Palao, Irene Carretero-Barrio, Sara Martos-Meléndez, Tamara Caniego-Casas, Marta Rosas, Noelia Martínez-Jañez, María Fernández-Abad, María Concepción Sánchez, Teresa Presa-Abos, Javier Zamora, Juan Martínez-Aedo Castro, María J Ledesma-Carbayo, José Palacios, Belén Pérez-Mies

**Affiliations:** ^1^ Department of Pathology, Severo Ochoa University Hospital, Madrid, Spain; ^2^ Faculty of Medicine, Alcalá University, Alcalá de Henares, Spain; ^3^ Department of Pathology, Ramón y Cajal University Hospital, Ramón y Cajal Health Research Institute (IRYCIS), Madrid, Spain; ^4^ Centre for Biomedical Research in Cancer Networks (CIBERONC), Carlos III Health Institute, Madrid, Spain; ^5^ Department of General Surgery, Ramón y Cajal University Hospital, Ramón y Cajal Health Research Institute (IRYCIS), Madrid, Spain; ^6^ Department of Medical Oncology, Ramón y Cajal University Hospital, Ramón y Cajal Health Research Institute (IRYCIS), Madrid, Spain; ^7^ Department of Gynecology, Ramón y Cajal University Hospital, Ramón y Cajal Health Research Institute (IRYCIS), Madrid, Spain; ^8^ Department of Radiology, Ramón y Cajal University Hospital, Ramón y Cajal Health Research Institute (IRYCIS), Madrid, Spain; ^9^ Clinical Biostatistics Unit, Ramón y Cajal University Hospital, Ramón y Cajal Health Research Institute (IRYCIS), Madrid, Spain; ^10^ Centre for Biomedical Research in Epidemiology and Public Health (CIBERESP), Madrid, Spain; ^11^ Biomedical Image Technologies, ETSI Telecomunicación, Universidad Politécnica de Madrid, Spain; ^12^ Centre for Biomedical Research in Bioengineering, Biomaterials and Nanomedicine (CIBER-BBN), Madrid, Spain

**Keywords:** MammaPrint, breast cancer, TILs, HER2-low

## Abstract

MammaPrint® refines risk stratification in early oestrogen receptor-positive, HER2-negative breast cancer, evolving from a binary to a four-tier classification (UltraLow-Risk, Low-Risk, High-Risk 1, and High-Risk 2). The relationship between routine histopathological features, immune infiltration, and genomic risk within this framework remains incompletely characterized in luminal disease. We retrospectively analysed 492 luminal breast carcinomas with available MammaPrint® results. Clinicopathological variables (including histological subtype, grade, Ki-67, hormone receptor expression, lymphovascular invasion (LVI), and HER2-low status) were recorded. Stromal tumour-infiltrating lymphocytes (TILs) were quantified according to the criteria of Salgado *et al.* and spatially categorized as immune-deserted, stromal-restricted, immune-excluded, or inflamed patterns. CD4 and CD8 infiltration was assessed by immunohistochemistry on tissue microarrays. Associations with binary and four-tier MammaPrint® categories were examined using multivariable models. High-Risk tumours (41%) were enriched for increased grade, Ki-67, and LVI and lower PR expression. In High-Risk tumours, inflamed spatial patterns were more frequent and median TIL levels were significantly higher compared with Low-Risk tumours (15% vs 5%, *P* < 0.001). CD4 and CD8 infiltration increased with genomic risk, and CD4 retained a modest but statistically significant association after adjustment for conventional pathological variables. In the four-tier model, UltraLow-Risk/Low-Risk tumours showed minimal TILs and were enriched for invasive lobular carcinoma, whereas High-Risk 1/High-Risk 2 tumours displayed progressively higher proliferative and immune features. No association was observed between HER2-low status and genomic risk. Immune infiltration parallels proliferative and genomic risk gradients in luminal breast cancer. These findings indicate that immune descriptors align with the genomic risk continuum, although their independent prognostic contribution beyond established genomic assays requires further evaluation.

## Introduction

Most oestrogen receptor-positive (ER+) and human epidermal growth factor receptor 2-negative (HER2−) breast carcinomas are diagnosed in the early stages (1). Further stratification into luminal A and luminal B subtypes is routinely performed, as luminal B tumours are associated with higher relapse risk. Current international guidelines ESMO (2) and NCCN (3) recommend the use of validated multigene assays to refine adjuvant therapy decisions in early-stage ER+/HER− breast cancer and safely omit chemotherapy in genomic low-risk patients. Among these, Oncotype DX and MammaPrint® are the most widely implemented and supported by level 1A evidence (1, 4, 5).

MammaPrint® (Agendia, the Netherlands) is an RNA-based assay that provides a binary risk classification independent of clinicopathological parameters (1, 6, 7). The categorization is based on the MammaPrint Prognostic Index (MPI), a score ranging from −1.000 to +1.000 derived from the expression levels of 70 genes and comparison to reference profiles. An MPI >0.000 is classified as Low-Risk, and an MPI ≤0.000 is classified as High-Risk. This index has been extensively validated in prospective studies, such as RASTER or MINDACT (6, 7, 8).

Recently, the binary system has evolved into a four-tier risk model refining genomic risk values. High-Risk is subdivided into High-Risk 2 (HR2) (−1.000 to −0.570) and High-Risk 1 (HR1) (−0.569 to 0.000), and Low-Risk is subdivided into Low-Risk (LR) (+0.001 to +0.355) and UltraLow-Risk (+0.356 to +1.000). Data from the FLEX registry and MINDACT show meaningful prognostic and therapeutic differences between these subgroups. HR2 tumours benefit from chemotherapy at nearly twice the absolute rate compared with HR1, while UltraLow-Risk cases display indolent biology, with an 8-year breast cancer-specific survival of 99.6%, allowing consideration of therapy de-escalation (9, 10, 11).

Despite the growing clinical use of MammaPrint®, few studies have explored its relationship with histopathological parameters. Most available data come from retrospective or secondary analyses with heterogeneous populations. Histological grade, progesterone receptor (PR) expression, and proliferation index (Ki-67) consistently correlate with MammaPrint® High-Risk results, although the magnitude and direction of associations vary across series. Other features such as lymphovascular invasion (LVI) or tumour size have been less consistently linked to genomic risk (9, 12, 13, 14, 15, 16, 17, 18, 19). Moreover, the histopathological characteristics across the recently proposed four-tier MammaPrint® categories remain unexplored.

In addition, the clinical importance of the HER2-low phenotype (immunohistochemistry 1+ or 2+ without amplification) has risen following the success of trastuzumab deruxtecan, and this category is frequent among luminal breast cancers evaluated with MammaPrint®. However, studies assessing the relationship between HER2-low status and genomic assays have yielded inconsistent results: some report no significant association (20, 21, 22, 23), whereas others describe the opposite (24, 25). Thus, the impact of HER2-low expression on genomic risk classification remains uncertain.

Tumour-infiltrating lymphocytes (TILs) are established as prognostic markers in triple-negative (TN) and HER2-positive breast cancer, but their role in luminal tumours is unclear. Evidence suggests that higher levels of TILs may reflect tumour aggressiveness, being associated with higher grade and elevated Ki-67 rather than having independent prognostic value (26, 27, 28, 29). Furthermore, recent studies indicate that not only the quantity but also the spatial distribution of TILs (e.g. heterogeneous infiltration or marginal accumulation) may hold biological significance, particularly in luminal B tumours (29, 30, 31). Nevertheless, their association with genomic profiles has been scarcely examined. To date, only one congress abstract has reported data linking TILs and MammaPrint® risk, while studies using other assays, such as Oncotype DX, also show higher levels of TILs in genomic high-risk tumours (32, 33, 34, 35). Furthermore, the characterization of the tumour–immune microenvironment beyond total TIL counts, characterizing the distribution of specific subsets such as CD4 and CD8 T cells, may further delineate the tumour microenvironment associated with distinct genomic risk strata (36).

Finally, invasive lobular carcinoma (ILC) accounts for approximately 15% of luminal breast cancers; however, the predictive and prognostic value of genomic assays in this subtype remains less defined, since most validation series have predominantly included invasive carcinoma of no special type (IC-NST). Reported genomic risk in ILC also differs by platform, being generally higher when using MammaPrint® (37, 38). Moreover, ILC typically exhibits lower TIL levels than IC-NST (39), although some studies suggest worse outcomes in ILC with higher TILs (40).

This study aimed to analyse the clinicopathological characteristics and TIL profiles, including their spatial distribution and immune subsets, across MammaPrint® Low-Risk and High-Risk luminal breast carcinomas, and to investigate the histopathological differences within the four-tier MammaPrint® classification.

## Methods

### Study population and clinicopathological data

Between January 2012 and December 2023, patients with a valid MammaPrint® result obtained in routine clinical practice were identified from the database of a tertiary hospital. Clinicopathological data were retrospectively retrieved from medical records, including MammaPrint® result and index (MPI), age, menopausal status, tumour size, histological grade and its components (glandular/ductal formation, nuclear grade, and mitotic count) according to the Nottingham system, pT and pN stage, LVI and perineural invasion (PNI), ER and PR status (both percentage and Allred score categorized as low (0–4), intermediate (5, 6), and high (7, 8)), and Ki-67 index and HER2 status in which the immunohistochemical score (0, 1+, 2+, 3+) has been routinely specified in our institution throughout the study period and afterwards classified as HER2-0 or HER2-low. Immunohistochemical staining for ER, PR, Ki-67, and HER2 was performed using validated Agilent/Dako reagents (clone EP1, clone PgR 636, clone MIB-1, and HercepTest™ pharmDx kit) and scored according to the ASCO/CAP guidelines using proper external controls.

In cases where ER, PR, Ki-67, or HER2 scores were not explicitly documented or were incomplete in the original report, available histological slides were centrally reviewed by dedicated breast pathologists and rescored according to contemporary ASCO/CAP guidelines to ensure consistent classification across the cohort.

### Histopathological review and TIL assessment

To ensure consistency, histological grade was re-evaluated on the same haematoxylin–eosin (H&E) slide used for MammaPrint® analysis, which also served for TIL evaluation following the criteria of Salgado *et al.* (41). TILs were assessed as the percentage of stromal area within the borders of the invasive tumour occupied by mononuclear inflammatory cells, excluding areas of ductal carcinoma *in situ*, necrosis, crush artefact, and regressive fibrosis. Results were recorded as continuous percentages.

**Figure 1:**
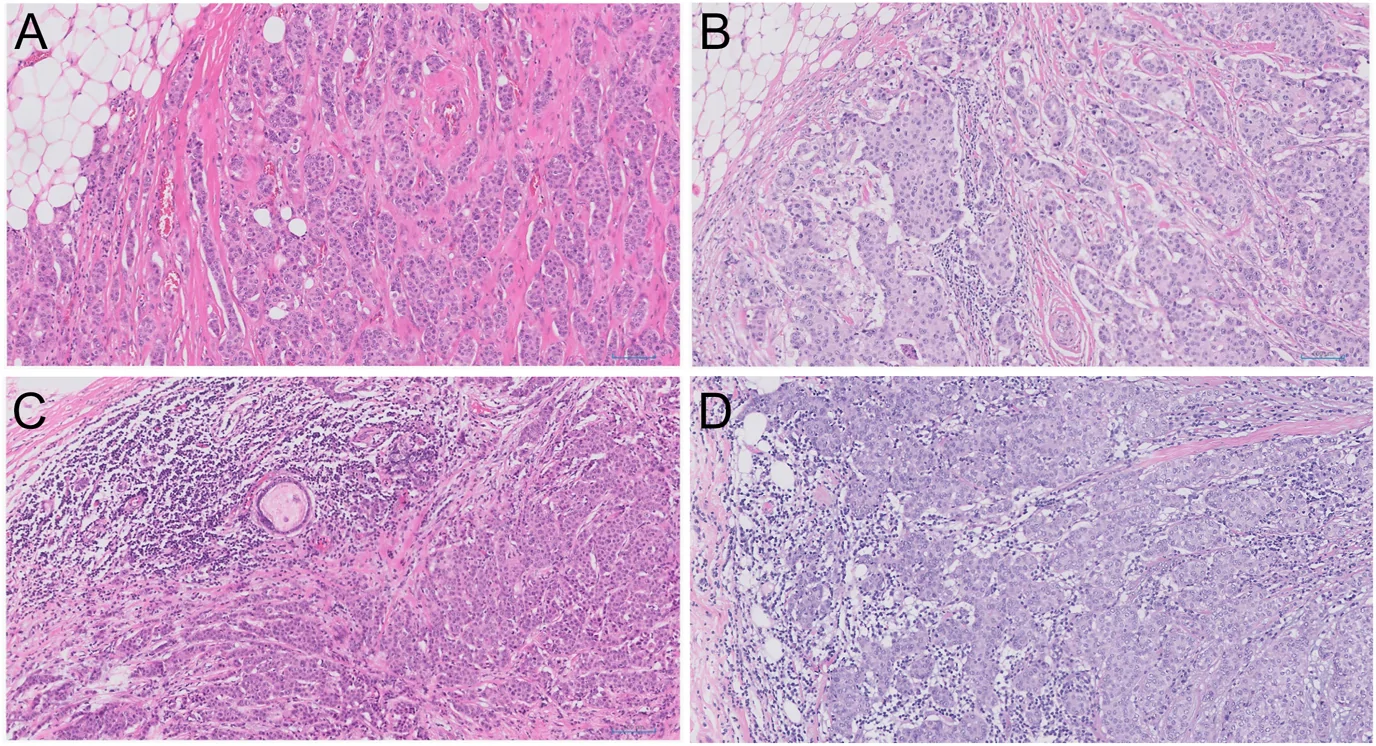
Representative histological images illustrating distinct patterns of tumour-infiltrating lymphocyte (TIL) distribution: (A) Immune-desert, characterized by absent or minimal stromal TILs (≤1%) within the invasive tumour area. (B) Stromal pattern, with lymphocytes predominantly confined to the intratumoural stroma and without prominent accumulation at the tumour–stroma interface. (C) Immune-excluded, showing dense lymphocytic infiltrates localized at the tumour–stroma interface with limited penetration into tumour cell nests. (D) Inflamed pattern, defined by the concurrent presence of lymphocytes within the tumour stroma and at the invasive tumour border. Haematoxylin and eosin (H&E), original magnification ×10. Scale bar: 100 μm.

In addition to quantitative assessment, spatial immune patterns were categorized based on previously described tumour–immune phenotypes originally defined using CD8 immunohistochemistry in triple-negative breast carcinoma (42). In this study, these categories were adapted for routine H&E evaluation, given the morphological nature of our assessment. Classification was based on the predominant architectural pattern as follows (Fig. 1): i) Immune-deserted tumours showed ≤1% stromal TILs. ii) Immune-excluded tumours displayed lymphocytic infiltrates predominantly localized at the tumour–stroma interface with minimal stromal permeation. iii) Stromal-restricted tumours exhibited lymphocytes confined to the intratumoural stroma without prominent accumulation at the invasive border. iv) Inflamed tumours were defined by the concurrent presence of lymphocytes within the tumour stroma and at the invasive tumour border.

All cases were independently reviewed by dedicated breast pathologists blinded to genomic risk results. In cases of discrepancy, a consensus evaluation was performed.

### Immunohistochemistry for CD4 and CD8

To further characterize the tumour microenvironment, immunohistochemical (IHC) staining for CD4 and CD8 was performed on tissue microarrays (TMAs) from 214 cases, each represented by two 1.5 mm cores selected from representative tumour areas, without consideration of the presence or density of TILs. Sections were processed on a Leica Bond automated platform using CD4 (clone CD4-368-L-CE) and CD8 (clone 4B11-L-CE) ready-to-use antibodies, with DAB detection (Bond Polymer Refine Kit, Leica). Representative TMAs are presented in Supplementary Figure 1 (see section on Supplementary materials given at the end of the article).

Quantification of CD8 and CD4 lymphocytes was restricted to the tumour-associated stromal compartment used for TIL assessment. Lymphocytes located within tumour cell nests, areas of ductal carcinoma *in situ*, necrosis, or artefact were excluded from evaluation.

For each core, the absolute number of positive cells within the evaluable stromal area was recorded. To account for potential intratumoural heterogeneity inherent to TMA sampling, both the mean value across the two cores and the maximum core value were calculated per case and used for statistical analyses.

As counts were expressed as absolute stromal cell numbers per TMA core and not as area-normalized densities (cells/mm^2^), results should be interpreted as core-based measures and may be influenced by sampling variability, so they may not reflect the full tumour spatial heterogeneity.

### Clinical outcome assessment

The clinical outcome of participants was measured according to the Standardized Definitions for Efficacy End Points (STEEP) criteria (43). Breast cancer-free interval (BCFI) was calculated from surgery to any breast cancer-related event (local, distant recurrence, or breast cancer death) and breast cancer-specific survival (BCSS) from surgery to death due to breast cancer. Follow-up was updated in June 2024.

### Statistical methods

Statistical analyses were performed using SPSS version 25.0 (IBM Corp., Armonk, NY, USA). Comparisons between binary and four-tier MammaPrint® risk groups and clinicopathological variables employed Chi-square tests for categorical data and Mann–Whitney U or Kruskal–Wallis tests for continuous variables. Significant findings underwent Holm-adjusted post-hoc pairwise comparisons. Variables significant in univariate analyses were included in multivariable binary logistic regression models to identify factors associated with high genomic risk, expressed as odds ratios (OR) with 95% confidence intervals (CI).

Correlations between continuous MPI values and TIL, CD4+, and CD8+ levels were assessed using Spearman’s rank coefficient (ρ). Collinearity among covariates included in multivariable models was evaluated using variance inflation factors (VIF). Due to strong collinearity when stromal TILs and CD4 were entered simultaneously in the same model, these variables were analysed in separate multivariable models. Model discrimination was assessed by the area under the receiver operating characteristic curve (AUC).

The association of TILs with histopathological characteristics was evaluated by univariate and multivariate linear regression, presenting unstandardized beta coefficients (B) and *P*-values. Two-sided *P*-values <0.05 were considered statistically significant.

## Results

### Baseline characteristics

A total of 492 patients with valid MammaPrint® results (2012–2023) were analysed. Of these, 202 (41.1%) were High-Risk and 290 (58.9%) were Low-Risk. According to the four-tier classification, 4.3% were HR2, 36.4% HR1, 48.4% Low-Risk, and 10.9% UltraLow-Risk. MPI was unavailable in six cases.

Median age was 56 years (IQR 49–65), and four patients (0.8%) were male. The median tumour size was 1.5 cm (IQR 1.2–2.0). Histological types included 76.8% IC-NST, 17.9% ILC, and 5.3% invasive carcinoma of special subtypes (IC-ST). The proportion of High-Risk results varied by subtype (44% IC-NST, 27% ILC, 38% IC-ST). Grade 2 tumours predominated (65%); most tumours were pT1c (62%) and node negative (73%). Detailed data are shown in Table 1.

**Table 1:** Association between clinicopathological variables and genomic results. Percentages are calculated among the total High-Risk or Low-Risk results for each category.

Categories	Total		Low-Risk		High-Risk		*P*-value
	N or Median	% or IQR	N or Median	% or IQR	N or Median	% or IQR	
Age (*n* = 491)	56	49.0–65.0	57	50.0–65.0	56	47.38–64.15	0.324
Menopausal status (N = 491)							0.700
No	188	38.3%	109	37.6%	79	39.3%	
Yes	303	61.7%	181	62.4%	122	60.7%	
Histological subtype (N = 492)							**0.012**
IC–NST	378	76.8%	210	72.4%	168	83.2%	
ILC	88	17.9%	64	22.1%	24	11.9%	
IC–ST	26	5.3%	16	5.5%	10	5.0%	
Tumour size cm (N = 489)	1.5	1.2–2.0	1.5	1.2–2.0	1.7	1.2–2.1	0.258
pT category (N = 491)							0.469
pT1a	3	0.6%	1	0.3%	2	1%	
pT1b	68	13.8%	40	13.8%	28	13.9%	
pT1c	305	62.1%	186	64.4%	119	58.9%	
pT2	115	23.4%	62	21.5%	53	26.2%	
pN category (N = 483)							0.323
pN0	361	74.7%	206	72.3%	155	78.3%	
pN1mi	81	16.8%	52	18.2%	29	14.6%	
pN1	41	8.5%	27	9.5%	14	7.1%	
Nottingham histological grade (N = 492)							**<0.001**
Grade 1	90	18.3%	74	25.5%	16	7.9%	
Grade 2	320	65%	198	68.3%	122	60.4%	
Grade 3	82	16.7%	18	6.2%	64	31.7%	
Tubule formation (N = 482)							**0.005**
Score 1	45	9.3%	36	12.7%	9	4.5%	
Score 2	136	28.2%	82	29%	54	27.1%	
Score 3	301	62.4%	165	58.3%	136	68.3%	
Nuclear grade (N = 483)							**<0.001**
Score 1	10	2.1%	9	3.2%	1	0.5%	
Score 2	241	49.9%	178	62.7%	63	31.7%	
Score 3	232	48%	97	34.2%	135	67.8%	
Mitotic index (N = 482)							**<0.001**
Score 1	361	74.8%	247	87.3%	114	57.3%	
Score 2	91	18.9%	30	10.6%	61	30.7%	
Score 3	30	6.2%	6	2.1%	24	12.1%	
Nottingham grade total score (N = 484)	6	6.0–7.0	6	5.0–6.0	7	6.0–8.0	<0.001
Lymphovascular invasion (N = 485)							**0.010**
Absent	427	88%	260	91.2%	167	83.5%	
Present	58	12%	25	8.8%	33	16.5%	
Perineural infiltration (N = 486)							0.154
Absent	417	85.8%	240	83.9%	177	88.5%	
Present	69	14.2%	46	16.1%	23	11.5%	
Ki67 percentage (N = 488)	13	8.0–20.0	10	5.0–15.0	18	10.0–25.0	**<0.001**
ER percentage (N = 488)	90	80–100	90	80–100	90	80–100	**0.136**
ER Allred score (N = 486)							**0.798**
Low (Allred 0–4)	4	0.8%	3	1.1%	1	0.5%	
Medium (Allred 5–6)	38	7.8%	22	7.7%	16	8%	
High (Allred 7–8)	444	91.4%	260	91.2%	184	91.5%	
PR percentage (N = 488)	40	5.0–80.0	50	10.0–90.0	30	0.0–70.0	**<0.001**
PR Allred score (N = 485)							**0.004**
Low (Allred 0–4)	157	32.4%	81	28.4%	76	38%	
Medium (Allred 5–6)	125	25.8%	67	23.5%	58	29%	
High (Allred 7–8)	203	41.9%	137	48.1%	66	33%	
HER2 (N = 486)							0.864
HER2-0	151	31.1%	88	30.8%	63	31.5%	
HER2-low	335	68.9%	198	69.2%	137	68.5%	

IC-NST: Invasive carcinoma, no special type. ILC: Invasive lobular carcinoma. IC-ST: Invasive carcinoma of special subtypes. ER: Oestrogen receptor. PR: Progesterone receptor.

### Association between MammaPrint® and clinicopathological factors

MammaPrint® High-Risk results were significantly associated with IC-NST histology, grade 3 tumours, LVI, low PR expression, and higher Ki-67 (see Table 1).

### Association between MammaPrint® result and tumour microenvironment

TIL evaluation was available for 482 cases. Median TILs were 5% (IQR 3–15%). Overall, the most prevalent immune phenotypes were immune-excluded and immune-deserted (32 and 30.3%). High-Risk tumours displayed significantly higher TIL levels than Low-Risk (15 vs 5%, *P* < 0.001). The distribution of immune patterns differed significantly according to MammaPrint® risk classification (*P* < 0.001): immune-deserted predominated in Low-Risk tumours, while immune-inflamed patterns were enriched in High-Risk cases (Table 2).

**Table 2:** Association between MammaPrint® risk classification and tumour–immune microenvironment. Percentages are calculated within each risk group for each category.

Categories	Total		Low-Risk		High-Risk		*P*-value
	N or Median	% or IQR	N or Median	% or IQR	N or Median	% or IQR	
TILs – continuous (N = 482)	5	3.0–15.0	5	3.0–10.0	15	4.5–20.0	<0.001
TIL distribution (N = 475)							<0.001
Immune-deserted	144	30.3%	99	34.7%	45	23.7%	
Stromal-restricted	120	25.3%	84	29.5%	36	18.9%	
Immune-excluded	152	32.0%	84	29.5%	68	35.8%	
Inflamed-pattern	59	12.4%	18	6.3%	41	21.6%	
CD4 and CD8 TIL profile (N = 214)							
CD4 mean (absolute numbers)	8	0.0–31.0	3	0.0–20.3	15.5	4.0–68.5	<0.001
CD4 max (absolute numbers)	12	0.0–50.0	6.0	0.0–30.3	27.0	8.0–98.0	<0.001
CD8 mean (absolute numbers)	25.5	10.0–45.0	20.0	10.0–35.0	32.5	12.0–65.0	0.0036
CD8 max (absolute numbers)	30.0	14.0–60.0	30.0	14.0–50.0	40.0	15.0–90.0	0.0038

TILs, tumour-infiltrating lymphocytes.

The immunohistochemistry study carried out in 214 cases in TMAs revealed higher CD4 and CD8 infiltration in High-Risk tumours (*P* < 0.001 and *P* = 0.0036, respectively) (Table 2 and Supplementary Figure 2).

Spearman’s correlation analysis demonstrated a modest but statistically significant negative association between continuous MPI values and continuous TIL levels (ρ = −0.239, *P* < 0.001; *n* = 478), with CD4 (ρ = −0.360 for mean and −0.368 for max CD4 count) and with CD8 (ρ = −0.238 for mean and −0.236 for max CD8 count; all *P* < 0.05). CD4 showed the strongest association.

Collinearity diagnostics confirmed a strong overlap between TILs and CD4 (VIF > 7), whereas CD8 was independent (VIF < 2). Therefore, TILs and CD4 were analysed in separate multivariable models.

### Association between TILs and clinicopathological factors

In univariate linear regression, higher TIL infiltration correlated with histological grade (B = 4.47, *P* < 0.001), nuclear pleomorphism (B = 5.76, *P* < 0.001), LVI (B = 4.28, *P* = 0.006), MammaPrint® High-Risk status (B = 6.61, *P* < 0.001), and Ki-67 index (B = 0.26, *P* < 0.001); while age was inversely related (B = −0.11, *P* = 0.023). In multivariable analysis, grade, Ki-67, and genomic risk remained independent predictors; age and LVI lost significance.

### Predictors of MammaPrint® High-Risk classification

A multivariable logistic regression model including TILs, PR, Ki-67, and Nottingham score was constructed to identify factors associated with genomic high-risk classification.

Collinearity diagnostics were performed for all covariates included in the model. Variance inflation factor (VIF) values remained within acceptable limits for Nottingham score (VIF 1.51), Ki-67 (1.49), PR (1.00), and TILs (1.07), indicating the absence of relevant multicollinearity. When TILs and CD4 were entered simultaneously in the same model, strong overlap was observed (VIF >7); therefore, these variables were analysed in separate multivariable models.

Model discrimination was acceptable, with an area under the receiver operating characteristic curve (AUC) of 0.78 for the model including TILs.

In the adjusted model, the Nottingham total score was the strongest determinant, with each unit increase raising the odds of High-Risk status by 54%. Ki-67 and TILs showed modest positive associations (OR: 1.08 and 1.05), whereas PR was inversely associated (OR: 0.99) (Table 3).

**Table 3:** Multivariable binary logistic regression analysis of factors associated with high genomic risk. Odds ratios are shown with 95% confidence intervals (CI).

Variable	Odds ratio	95% CI	*P*-value
TILs	1.048	1.024–1.072	<0.001
Nottingham total score	1.539	1.222–1.937	0.00024
PR percentage	0.990	0.984–0.996	0.0016
Ki67	1.082	1.050–1.116	<0.001
Constant	0.012	0.003–0.049	<0.001

TILs, tumour-infiltrating lymphocytes.

When analysed by histologic subtype, TILs remained statistically associated with High-Risk status only in IC-NST (OR: 1.048; 95% CI: 1.021–1.075; *P* < 0.001).

Replacing TILs with CD4 and CD8 in the model, the Nottingham total score remained significantly associated with genomic high-risk classification (OR: 1.588; 95% CI: 1.069–2.361; *P* = 0.022), together with Ki-67 (OR: 1.065; 95% CI: 1.019–1.113; *P* = 0.005) and PR (OR: 0.990; 95% CI: 0.981–0.999; *P* = 0.029). Among immune markers, only CD4 retained statistical association with High-Risk classification (OR: 1.019; 95% CI: 1.005–1.033; *P* = 0.006), whereas CD8 was not significantly associated (OR: 0.996; 95% CI: 0.986–1.005; *P* = 0.369).

The model including CD4 and CD8 showed similar discriminative performance (AUC: 0.79).

### Clinicopathological features across the four-tier MammaPrint® groups

When patients were stratified into the four MammaPrint® risk categories (UltraLow-Risk, Low-Risk, HR1, and HR2), age, menopausal status, and tumour size did not differ significantly, although HR2 tumours tended to be larger (*P* = 0.068).

**Figure 2:**
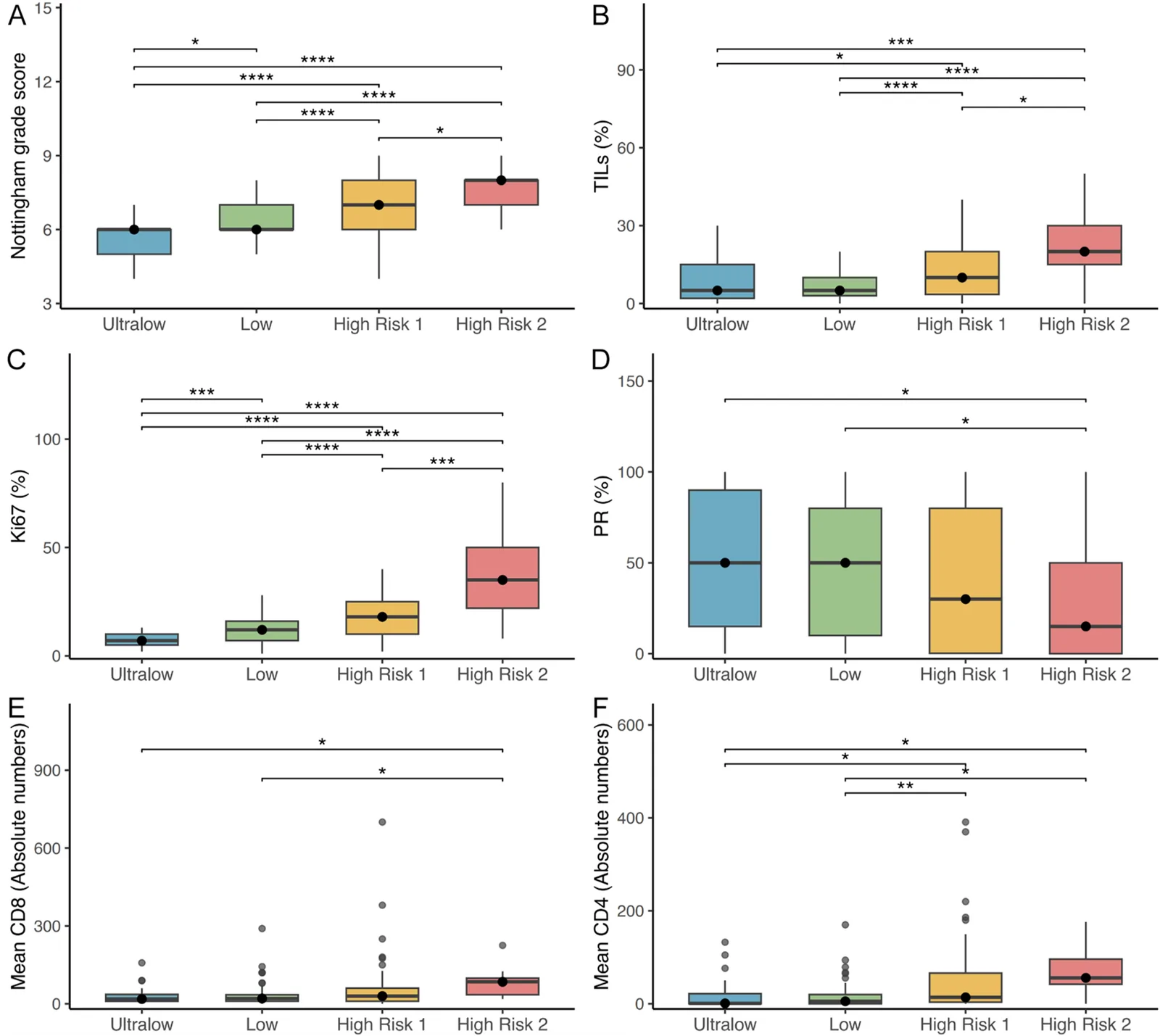
Boxplots illustrating the distribution of (A) Nottingham histological score, (B) tumour-infiltrating lymphocytes (TILs), (C) Ki-67, (D) progesterone receptor (PR) expression, (E) mean CD8 count and (F) mean CD4 count across MammaPrint risk groups. Overall differences were assessed using the Kruskal–Wallis test. Pairwise comparisons were performed using Mann–Whitney U tests with Holm adjustment for multiple testing; significance is indicated by brackets and asterisks (*P ≤ 0.05; **P ≤ 0.01; ***P ≤ 0.001; ****P ≤0.0001). Non-significant comparisons are not displayed.

Histological subtype was significantly associated with genomic risk. ILC and tubular carcinomas predominated in the UltraLow-Risk group, whereas ILC was uncommon in HR1 and absent in HR2. Histological grade varied markedly across categories: UltraLow-Risk and Low-Risk tumours were mainly grade 1 and 2, while HR1 and HR2 were enriched in grade 3. The Nottingham total score increased progressively from UltraLow-Risk to HR2 (all post-hoc *P* < 0.05 except UltraLow-Risk vs Low-Risk; Fig. 2A).

Lymphovascular invasion (LVI) also differed significantly across categories (*P* = 0.039), largely reflecting its higher frequency in HR1 compared with Low-Risk tumours (*P* = 0.024).

TIL levels were strongly associated with genomic risk (*P* < 0.001). UltraLow-Risk and Low-Risk tumours consistently showed low lymphocytic infiltration, whereas HR1 and HR2 categories demonstrated progressively higher median TIL levels. Post-hoc analyses confirmed significantly higher TILs in HR1/HR2 compared with UltraLow-Risk/Low-Risk (all *P* < 0.001; Fig. 2B). CD4 and CD8 counts likewise showed increasing values across risk categories (CD8 *P* = 0.004–0.007 for maximum and mean values; CD4 *P* < 0.001; Fig. 2E and F).

Proliferation assessed by Ki-67 was significantly associated with risk group (*P* < 0.001). Median Ki-67 values were 10% in UltraLow-Risk and Low-Risk, 20% in HR1, and 30% in HR2, with significant pairwise differences between categories except UltraLow-Risk versus Low-Risk after Holm correction (Fig. 2C and Supplementary Table 1).

Continuous ER percentage did not differ significantly across categories (*P* = 0.117), although Allred ER scores showed statistical differences (*P* < 0.001), with UltraLow-Risk/Low-Risk tumours predominantly displaying uniformly high scores (Allred = 8) and HR2 enriched for relatively lower values. PR percentage also differed across groups (*P* = 0.005), with lower levels observed in HR1/HR2, although post-hoc significance was not maintained after multiple comparison correction (Fig. 2D). HER2-low status did not vary significantly among categories (*P* = 0.460).

Overall, the four-tier MammaPrint® categories parallelled progressive differences in histological grade, proliferation index (Ki-67), immune infiltration (TILs and CD4/CD8) and histologic subtype; whereas other clinicopathological parameters displayed more homogeneous distributions across groups (Supplementary Table 1).

### Breast cancer-free interval and MammaPrint® classification

Patients were followed for a total of 8,097.5 patient-years, with a median follow-up of 9.2 years (IQR: 5.6–17.4). Seven patients (1.4%) were lost to follow-up. Twenty-two breast cancer-related events occurred (4.5%): 11 local recurrences, 10 distant metastases, and one combined event. Recurrences were distributed as follows: 15 in the Low-Risk group, 6 in HR1, 1 in HR2, and none in the UltraLow-Risk group. Four patients (0.8%) died of breast cancer, all following distant recurrence and classified as HR1.

Systemic treatment decisions were made in routine clinical practice and were guided by the MammaPrint® genomic risk result, in accordance with contemporary clinical guidelines. Given the limited number of events and deaths, and the non-randomized, treatment-influenced design of the cohort, survival analyses were considered exploratory.

Breast cancer-free interval (BCFI) did not differ significantly between binary MammaPrint® categories (log-rank *P* = 0.856), and median BCFI was not reached in either group.

When analysed according to the four-tier classification, no statistically significant differences in BCFI were observed (log-rank *P* = 0.280). Although HR2 cases showed numerically shorter BCFI compared with Low-Risk tumours, this did not reach statistical significance (*P* = 0.059).

## Discussion

Gene-expression assays are increasingly used to guide adjuvant chemotherapy in early-stage ER+/HER2-negative breast cancer (1, 2, 3). Among them, MammaPrint® provides both binary and four-tiered genomic risk classifications (6, 8, 9). This study explored the relationship between MammaPrint® risk groups and histopathological features in a large series of luminal breast carcinomas, with a special focus on the tumour microenvironment, an area less extensively investigated.

In our series, MammaPrint® High-Risk tumours showed higher Nottingham grade, increased Ki-67, lower PR, more frequent LVI, and predominance of IC-NST over ILC/IC-ST. These findings align with other studies, where genomic high risk correlated strongly with grade and proliferation; whereas associations with PR, LVI, or histological subtype tend to be weaker (12, 13, 14, 16, 17, 18, 19). Histological grade derives from the Nottingham total score, a continuous sum of three components (44). While typically collapsed into three categories, using the value of continuous Nottingham total score may better preserve information. This has been adopted in the Magee Equations for Oncotype DX, and Bedell *et al.* have reported strong correlations of both MammaPrint® and Magee with the Nottingham score (18, 45).

Consistently, we observed progressive shifts in median Nottingham scores across the four MammaPrint® categories, which supports the view that the continuous score aligns with genomic risk and may refine decisions on when genomic testing is indicated (45, 46, 47, 48, 49).

The clinical relevance of HER2-low has risen with trastuzumab deruxtecan. Most luminal tumours undergoing genomic testing fall into this category (25). However, few studies have assessed the significance of this feature in the context of multigene assays, with conflicting results. In our cohort, HER2-low tumours showed no association with MammaPrint® risk classifications, aligning with Zhang *et al.* (21) and Sivapiragasam *et al.* (23), both including the four-tier classification. Conversely, Baez-Navarro *et al.* (25) observed a slightly higher proportion of MammaPrint® High-Risk cases among HER2-low compared with HER2-0 in luminal patients (39 versus 34%, *P* < 0.049). Similar inconsistencies appear with other assays, like Oncotype DX or Prosigna/PAM50 (22, 24, 50). These divergences likely reflect inter-observer variability in HER2-low IHC scoring, differences in assay composition and gene weighting, variation in sample size, and the likelihood that HER2-low represents a biological continuum rather than a discrete subgroup (51, 52, 53).

Consistent with this, we observed no differences in TIL levels between HER2-low and HER2-0 (data not shown), mirroring Fernandes *et al.* (54), and contrasting with the well-known immune enrichment of HER2-positive disease.

In triple-negative and HER2-positive cancers, high TILs are associated with better outcomes and therapy responsiveness (1, 28). In luminal disease, however, data are heterogeneous. A pooled analysis of 3,771 cases showed higher pathological complete response (pCR) rates with increasing TILs across subtypes, but in luminal tumours, better overall survival correlated with lower TILs (27). A meta-analysis of 33 studies similarly found higher TILs to be linked to shorter overall survival in luminal disease and no clear association with pCR (26). Higher TILs in luminal tumours are associated with higher grades and Ki-67, suggesting they reflect aggressive biology rather than independently favourable immunity (28, 29).

Our findings are concordant with this model. In univariate analyses, higher grade, nuclear pleomorphism, LVI, Ki-67, and High-Risk status were associated with increased TILs, while older age correlated with lower infiltration. In multivariable models, grade, Ki-67, and genomic risk remained independently associated with TIL density, while age and LVI lost significance. Furthermore, High-Risk tumours harboured higher TIL levels (median 15 vs 5%) and showed a shift towards inflamed spatial phenotypes. To date, only one congress abstract has directly linked TILs and MammaPrint®, also reporting higher TILs in High-Risk cases. However, this series is smaller, and no cut-off point for high TILs was reported (35).

Evidence relating to TILs and Oncotype DX is broader. Tong *et al.* (33) quantified TILs in 385 digitized whole slides, classifying them as low (≤10%) or high (>10%), and evaluated CD3, CD4, CD8, and PD-L1 expression. Consistent with our findings, higher TIL levels were associated with higher recurrence scores, higher grade, lower PR, and increased Ki-67. They also observed that tumours with high recurrence scores more often displayed an inflamed phenotype, suggesting that the spatial organization of TILs may have prognostic relevance. The localization of lymphocytes within the tumour microenvironment (stromal versus intratumoural) may influence clinical behaviour, including in ER-positive disease (55). The inflamed spatial pattern has been associated with increased immune activation. Conversely, the immune desert phenotype has been linked to poor immune surveillance and limited benefit from chemotherapy or immunotherapy (28, 36, 56). These data suggest that in ER+/HER2-negative disease, immune activation signals aggressive tumour biology and potential endocrine resistance.

Ahn *et al.* (32) and Kolberg-Liedtke *et al.* (34) reported higher Oncotype DX scores in tumours with increased TILs, although correlations were weak and lacked independent prognostic value in ER+/HER2-negative disease. Similarly, our correlation between MPI and TILs was moderate but significant. Unlike those studies, TILs in our analysis remained an independent predictor of MammaPrint® High-Risk classification after adjusting for PR, Ki-67, and histological grade, suggesting that MammaPrint® risk classification reflects aspects of the tumour–immune microenvironment. This association, however, should be interpreted as biologically informative rather than clinically actionable, as TILs did not add independent prognostic value beyond standard clinicopathological variables in our cohort.

Extending the analysis to immune subsets, both CD4 and CD8 stromal infiltrates correlated inversely with MPI, with stronger associations for CD4. In multivariable models including conventional pathological variables, CD4 retained statistical association with genomic high-risk classification. However, effect sizes were modest, and CD4 represents a biologically heterogeneous compartment. Therefore, these findings should be interpreted cautiously and primarily as evidence that aspects of the adaptive immune microenvironment parallel genomic high-risk biology in luminal breast cancer. In addition, Tong *et al.* (33) reported stronger correlations between CD8 and the 21-gene recurrence score, while CD4 did not reach independent significance. Such discrepancies may reflect differences in the genomic platforms evaluated, or the methodology of immune quantification, and highlight the heterogeneity of immune signatures in ER+/HER2-negative breast cancer. Limited data specifically testing CD4 in luminal disease support biological plausibility. Moura *et al.* (36) observed increased CD4 in luminal B vs luminal A, consistent with a more immunosuppressive microenvironment.

Stratified models by histology revealed that TILs are independently associated with High-Risk status only in IC-NST; in ILC, this effect disappeared and Ki-67 was the sole significant variable. This is consistent with the lower immunogenicity of ILC (39, 40).

Across studies, the prevalence of High-Risk results in ILC has been consistently lower than in IC-NST (14, 57). In our cohort, 27% of ILC were High-Risk, comparable with the 26.7% reported by Beumer *et al.* (37), but higher than the 11% by Jenkins *et al.* (58). The MINDACT sub-analysis (487 ILC) confirmed that MammaPrint® retains prognostic value in ILC (16.2% High-Risk vs 39.1% IC-NST) with comparable outcomes across genomic strata (38).

In our series, four-tier stratification clarified biological gradients that were less apparent in the binary model. UltraLow-Risk/Low-Risk tumours were enriched for lobular/tubular histology, low-grade Nottingham scores, minimal TILs, and very low Ki-67, while HR1/HR2 showed progressively higher grade/Nottingham total score, more vascular invasion, higher Ki-67, and higher TILs with inflamed patterns. No recurrences occurred in the UltraLow-Risk group. These observations echo MINDACT (11, 59), FLEX (9), and the IKA trials (60). At the opposite extreme, HR2 tumours appear more proliferative and chemosensitive (9). A trend towards shorter BCFI was also observed in the HR2 group.

Notably, 70% of recurrences occurred in the Low-Risk group. However, the higher number of recurrences in the Low-Risk group likely reflects its larger sample size and the treatment-guided design of the cohort, rather than intrinsic biological behaviour, and should therefore be interpreted cautiously.

In conclusion, beyond classical variables such as Ki-67, histological grade, and PR, both TILs and CD4 were significantly associated with genomic high-risk classification and may help contextualize genomic results within routine histopathological assessment. Higher TILs correlated with genomic high risk, supporting their link to more aggressive biology, although their prognostic value remains uncertain due to the limited number of events. Larger prospective studies with whole-slide immune profiling and genomic validation are warranted to confirm these findings in ER+/HER2− breast cancer.

This study has limitations, including its retrospective single-centre design, potential selection bias, and CD4 and CD8 assessment on TMAs rather than whole slides. Although cores were sampled from representative tumour areas, this approach may underestimate the spatial heterogeneity of TILs across the tumour. However, its strengths lie in the large, well-characterized cohort, centralized pathology review, and integration of MammaPrint® results with detailed immune profiling.

## Supplementary materials







## Declaration of interest

Carmen Ariño-Palao reports that HealthInCode (distributor of MammaPrint**®** in Spain) covered her registration fee for the Spanish National Congress of Pathology in 2025. She declares no other competing interests. All remaining authors have declared no conflicts of interest.

## Funding

This study has been funded by Instituto de Salud Carlos III (ISCIII) through the project “PI25/01933” and ‘PI22/01892’ and co-funded by the European Union. This study was developed with the financial support of the Immune4ALL Project (PMP22/00054), with European funds of the Recovery, Transformation and Resilience Plan, and financed by the Instituto de Salud Carlos III. This work has also been funded by CIBERONC (grant CB16/12/00316) and Ministerio de Ciencia e Innovación, Agencia Estatal de Investigación10.13039/501100011033/MCIN/AEI/ERDF, under grants PDC2022-133865-I00 and PID2022-141493OB-I00 (10.13039/501100011033/MCIN/AEI/ERDF, UE), and co-financed by the European Development Regional Fund ‘A way to achieve Europe’ (ERDF).

## Compliance with ethical standards

All procedures complied with institutional and national ethical standards and the 1964 Declaration of Helsinki. The study was approved by the Ethics Committee of Hospital Ramón y Cajal (approval code 278-21). The requirement for informed consent was waived owing to the retrospective nature of the study and minimal material use.
