# Supplementary material for: Tumour-infiltrating lymphocytes differ across MammaPrint® classifications in breast cancer
Source: Endocr Relat Cancer. 2026 Sep 15;33(9):e260116. doi: 10.1530/ERC-26-0116 (PMC13615849; doi:10.1530/ERC-26-0116)
Supplement: Supplementary file 1 [file ERC-26-0116_supplementary_figure_1.pdf]

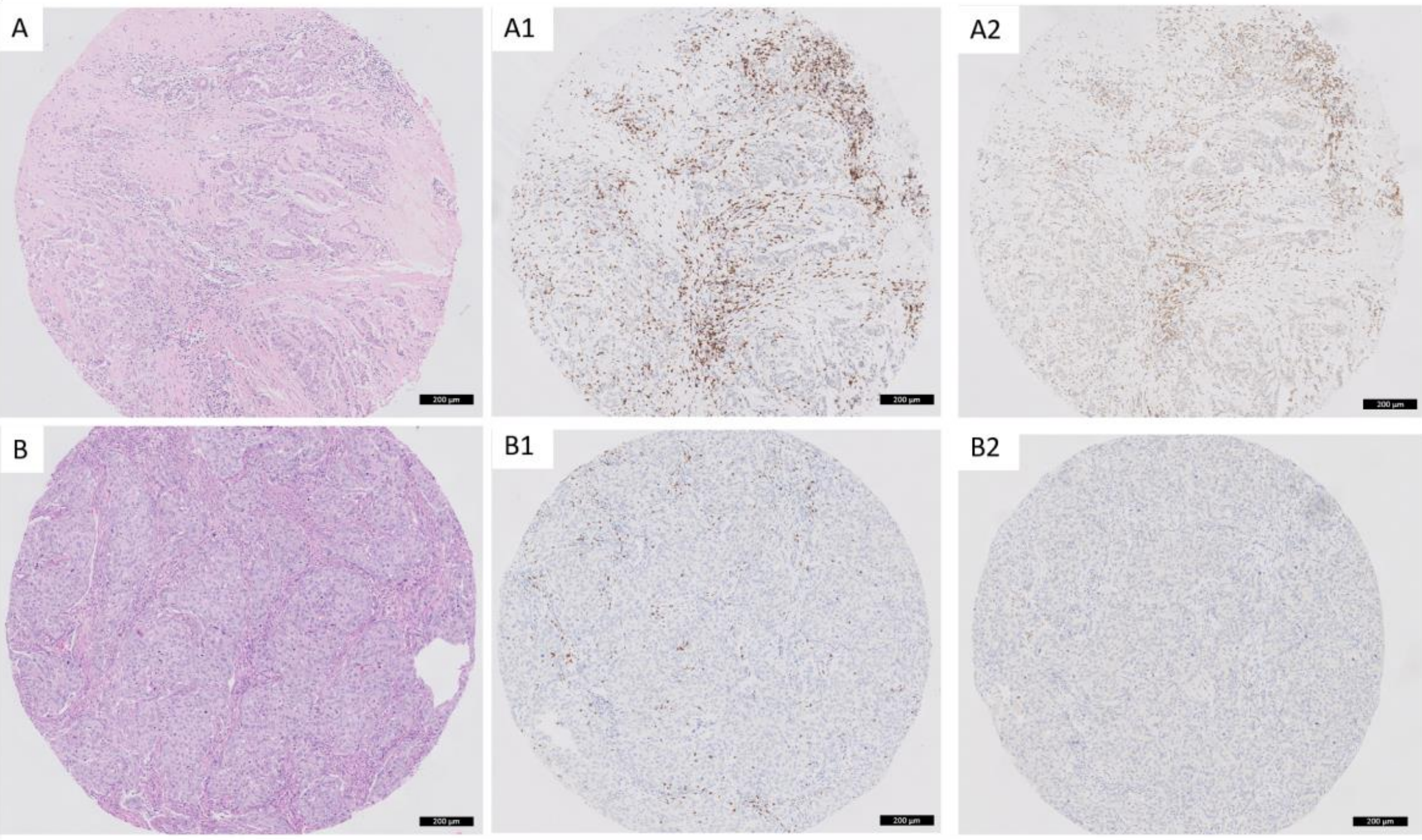
A
A1
A2
B
B1
B2
200 µm
200 µm
200 µm
200 µm
200 µm
200 µm
