# Supplementary material for: Tumour-infiltrating lymphocytes differ across MammaPrint® classifications in breast cancer
Source: Endocr Relat Cancer. 2026 Sep 15;33(9):e260116. doi: 10.1530/ERC-26-0116 (PMC13615849; doi:10.1530/ERC-26-0116)
Supplement: Supplementary file 2 [file ERC-26-0116_supplementary_figure_2.pdf]

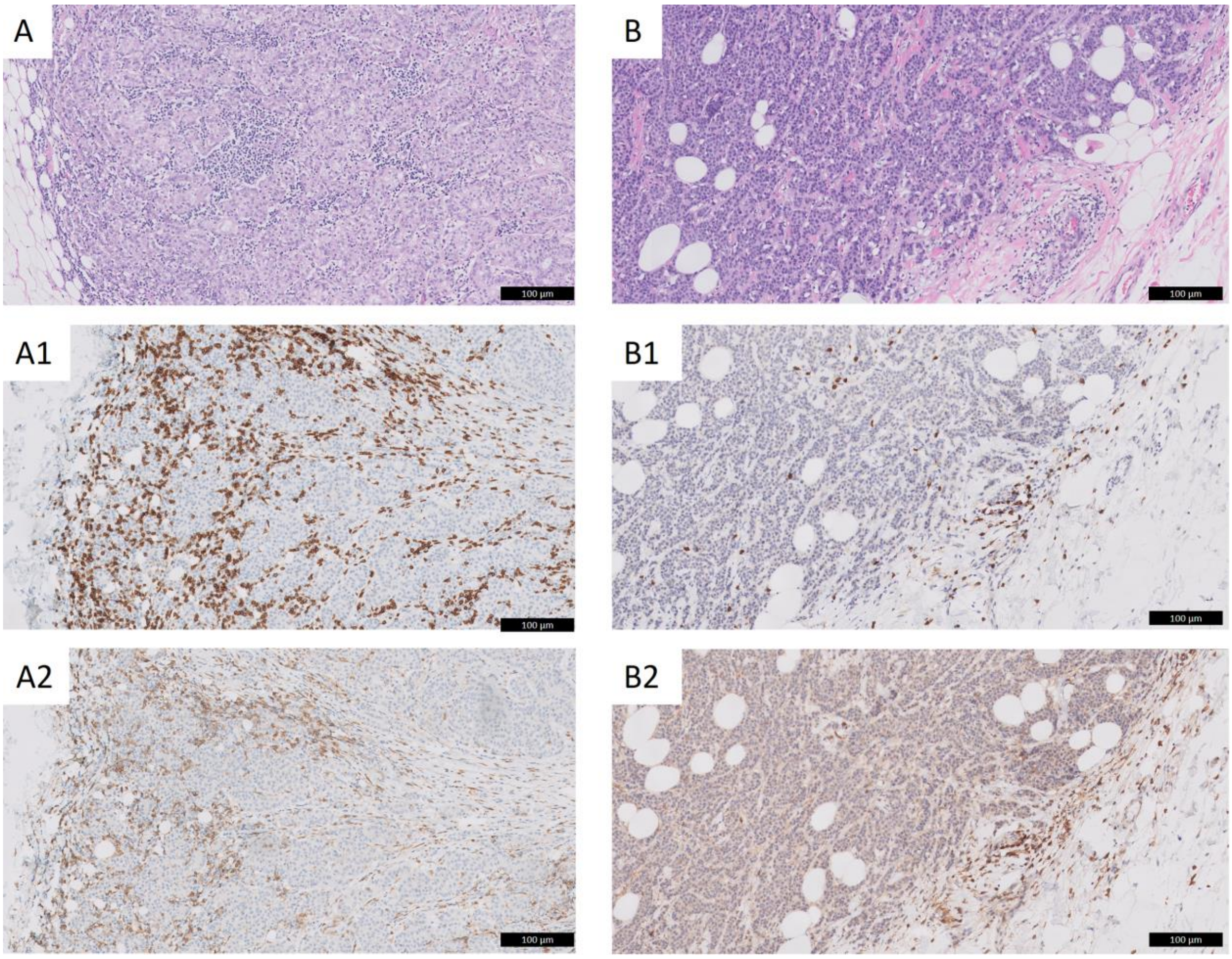
A
B
A1
B1
A2
B2
100 µm
100 µm
100 µm
100 µm
100 µm
100 µm
